# Determinants of Subjective Health, Happiness, and Life Satisfaction among Young Adults (18-24 Years) in Guyana

**DOI:** 10.1155/2020/9063808

**Published:** 2020-01-24

**Authors:** Chao Wang, Jiaxuan Liu, Run Pu, Zhifei Li, Wei Guo, Zhanchun Feng, Rui Huang, Bishwajit Ghose, Lu Ji, Shangfeng Tang

**Affiliations:** ^1^School of Public Policy and Management, China University of Mining and Technology, Xuzhou 221116, Jiangsu, China; ^2^School of Safety Engineering, China University of Mining and Technology, Xuzhou 221116, Jiangsu, China; ^3^Queen Mary School, Nanchang University, Nanchang 330006, Jiangxi, China; ^4^China National Center for Biotechnology Development, Beijing 100039, China; ^5^School of Medicine and Health Management, Tongji Medical College, Huazhong University of Science and Technology, Wuhan 430030, Hubei, China; ^6^Research Center for Rural Health Service, Key Research Institute of Humanities & Social Sciences of Hubei Provincial Department of Education, Wuhan 430030, Hubei, China; ^7^School of Pharmacy, Tongji Medical College, Huazhong University of Science and Technology, Wuhan 430030, Hubei, China; ^8^Faculty of Social Sciences, School of International Development and Global Studies, University of Ottawa, Ottawa, ON, Canada K1N 6N5

## Abstract

*Abstract*. Persistent urban-rural disparity in subjective health and quality of life is a growing concern for healthcare systems across the world. In general, urban population performs better on most health indicators compared with their rural counterparts. However, research evidence on the urban-rural disparity on perceived health, happiness, and quality of life among the young adult population is scarce in South American countries like Guyana. Therefore, in the present study we aimed to investigate whether subjective health, happiness, and quality of life differ according to place of residence among the young adult population in Guyana.

**Methods:**

Cross-sectional data on 2,434 men and women aging between 15 and 24 years were collected from the most recent Guyana Multiple Indicator Cluster Survey conducted in 2014. Outcome variables were perceived: satisfaction about health, life, and happiness, as well as life satisfaction before and after one year from the time of the survey. The urban-rural disparity in reporting satisfaction for these indicators was assessed by multivariate regression methods and by adjusting for relevant sociodemographic factors.

**Results:**

More than four-fifth of the respondents reported satisfaction with health (82.4%) and life (81.4%) and 77.9% reported being happy. A vast majority expressed improvement in life situation compared with a year ago (81.4%), and nearly all of the participants (95.4%) expect to have better life situation a year later. Multivariate analysis revealed an inverse association between rural residence and subjective health among men [OR = 0.518, 95%CI = 0.297, 0.901], and happiness [OR = 0.662, 95%CI = 0.381, 0.845] and life satisfaction [OR = 3.722, 95%CI = 1.502, 9.227] among women. Women having secondary [OR = 2.219, 95%CI = 1.209, 3.720] and higher [OR = 1.600, 95%CI = 1.041, 3.302] education also had higher odds of satisfaction with happiness.

**Conclusions:**

Our findings demonstrate the existence of significant urban–rural disparities in perceived health and quality of life among the young adult population in Guyana, especially among women. National health promotion projects should therefore take proper policy actions to address the underlying factors contributing to the urban–rural gaps in order to establish a more equitable healthcare system. Further researches are necessary to explore the underlying causes behind such disparities.

## 1. Introduction

Population health status has been shown to differ significantly across and within regions in both high- and low-middle income countries. The mechanisms that underlie the disparity are believed to be those surrounding the quality of food, air, and other living amenities, access to resources for income and education, and concentration of health centres [1–4]. Apart from the social and environmental determinants of health, variations in individual behavioural factors such as self-efficacy, hygiene, dietary, and healthcare seeking behaviour are also worthy of mentioning. Findings on urban-rural differences generated by epidemiological studies are usually designed to capture objective measures of health such as burden of morbidity and mortality, distribution of risk factors, use of healthcare facilities, as well as the subjective measures such as self-reported health, quality of life, and well-being [5–8].

There is a growing concern in the academia and civil society regarding the use of macroeconomic statistics, such as GDP, to portray the overall living conditions of a country. This is based on the consensus that although the ultimate objective of overall social and economic progress is to enhance happiness and quality of life, these subjective constructs have not received sufficient research and policy attention. Nonetheless, development researchers and economists are becoming increasingly interested in the concepts of psychological measures of well-being such as happiness and life satisfaction as a holistic indicator of national progress.

Review of the recent literature indicates that majority of the studies focus on objective measures of health, with a very little attention on the subjective measures, especially in the developing South American countries such as Guyana. This is understandable given the relatively lower popularity of the concept of and scarcity of data on subjective health in low-income settings. To this regard, we extracted data from UNICEF's Multiple Indicator Cluster Surveys on Guyana based on the availability of information on subjective measures of health. The purpose was to quantify and characterize differences in self-rated health, quality of life, and well-being between urban and rural adolescents and adults aged 15 to 49 years.

Urbanisation has been one of defining features of demographic transition in the developing and the transition economies and has been a topic of growing interest among health researchers [9, 10]. With the world's urban population surpassing the rural in recent years, there has been shifting of research focus to urban health. Although urbanisation generally embodies greater socioeconomic prosperity, health and living standards, some researchers argue that uncontrolled urban sprawl has led to increased concentration of poverty, poor sanitation and housing conditions, and neighbourhood safety which are associated with poor physical and mental health outcomes [11–13]. There is a general consensus regarding the facts that the beneficial effects of urban life are likely to be diminished by changing lifestyle factors that are associated with poor health outcomes: smoking and alcohol drinking, increased exposure to obesogenic environment, e.g., dependency on processed food, fewer opportunities for physical activity, environmental pollution, per capita availability of green space [14–17]. However, the distribution of the risk factors is likely to vary across and within countries and the relationship between health and the environmental factors is not necessarily a straightforward one. Although the population is predominantly rural and has the lowest proportion of urban population for any continents, Guyana is experiencing fast urbanisation with mounting challenges on health resources and environmental degradation [18–21]. While a low proportion of urban population can slacken development efforts, uncontrolled urban growth can create equally devastating consequences on population health and social stability that can eventually impede national prosperity. To this regard, studies on urban-rural differences on health and health determinants bear special importance to facilitate effective policy making and better allocation of resources for population health and development. 

## 2. Methods

Data for the present study were obtained from the UNICEF Multiple Indicator Cluster Survey conducted in 2014 (Guyana MICS round 5). The survey was carried out in by the Bureau of Statistics with technical support provided by the United Nations Children's Fund (UNICEF) and financial support by UNICEF, the Inter-American Development Bank (IDB), and the Government of Guyana. The objective of the survey was to generate quality data on a range of health and socioeconomic indicators to facilitate evidence-based policy making and to monitor progress towards the Millennium Development Goals (MDGs). MICS employs cluster sampling design to ensure representativeness of the data, including selection of enumeration areas across the countries, and then the second-stage selection of households from each of the enumeration areas for interview. Field work lasted from April to July 2014. In total 5,076 women (response rate of 87.4%) and 1,682 men (response rate 66.6%) aged 15–49 years were interviewed for the survey. However, the well-being module involving questions on subjective health, happiness, and life satisfaction was conducted only on young adults aged 15–24 years, which was the main inclusion criterion for this study. The other inclusion criterion was having provided the sociodemographic information included in the analysis.

### 2.1. Variables Used in the Study

Outcome variables: the outcome variables were self-reported satisfaction about (1) health (with the responses: Very Satisfied; Somewhat Satisfied; Neutral; Dissatisfied; Very dissatisfied), happiness (with the responses: Very happy; Somewhat happy; Neutral; Unhappy; Very unhappy), and life (with the responses: Very Satisfied; Somewhat Satisfied; Neutral; Dissatisfied; Very dissatisfied). Regarding life satisfaction, participants were further asked to rate the situation (1) one year ago (with the responses: Improved, More or less the same, Worsened) and (2) one year from now (with the responses: Better, More or less the same, Worsened). Thus, there were five outcome variables, all measured by respondent's own assessment. Similar approaches for measuring subjective health, happiness, and quality of life were used in a number of large-scale surveys including the Survey on Global Ageing and Adult Health (SAGE) and European Social Survey [22–24]. For this study, all the dependent variables were dichotomized in the following way: Satisfied (Very satisfied, somewhat satisfied) and Not satisfied (Neither satisfied nor unsatisfied, somewhat dissatisfied, Very dissatisfied); life satisfaction a year ago: Improved and Not improved (More or less the same, Worsened); (2) life satisfaction in a year from now: Better and Not better (More or less the same, Worsened).

### 2.2. Sociodemographic Indicators

A thorough literature review was conducted in PubMed to facilitate the selection of the explanatory variables that are theoretically relevant to the outcome measures. The predictors were selected based on the concepts of social determinants of health that regards health and well-being as a complex outcome of various social, economic, behavioural, and environmental issues. For instance, geographic disparity is a well-established determinant of health and access to resources that are in turn relevant to health outcomes. We have included the “region” variable to check whether participants in different regions report health and quality and life differently than others. Although the determinants of health and well-being are hard to encompass within a single model or framework, social research generally includes a combination of demographic, sociocultural factors to predict health and the risk factors. For this study, the choice of the variables was limited due to secondary nature of the data. Based on the availability from the survey, the following variables were included in this study: Age (15–19/20–24 years); Residency type (Urban/Rural); Sex (Men/Women); Currently married/in union (Yes/No); Residency (Urban/Rural); Region (Barima-Waini Pomeroon-Supenaam/ Essequibo Islands-West Demerara/ Demerara-Mahaica/ Mahaica-Berbice/ East Berbice-Corentyne/ Cuyuni-Mazaruni /Potaro-Siparuni/ Upper Takutu-Upper Essequibo Upper/ Demerara-Berbice; Education (None/ Secondary/ Higher); Ethnicity (East Indian/ African/ Amerindian/ Mixed Race); Wealth quintile (Poorest/ Second/ Middle/ Fourth/ Richest); User of TV/Radio (No/ Yes); User of Internet (Yes/No); Ever smokes (Yes/No); Ever drinks alcohol (Yes/No)[8, 22, 23, 25–30]. Wealth index (used in all MICS surveys) is calculated by using principal components analysis that involves assigning scores on individual household possessions, e.g., consumer goods, dwelling characteristics, water, and sanitation to generate factor scores for each item. Households are then ranked based on individual scores to range between poorest, poorer, middle, richer, and richest [31, 32].

### 2.3. Data Analysis

Data analyses were performed using Stata version 14. All analyses were weighted to account for the cluster survey design. Sample characteristics were presented by percentages. The predictors of each of the outcome measures were calculated using binary logistic regression techniques including the sociodemographic indicators that were associated with each of the outcomes at *p* < 0.1. Three separate multivariate models were run for each of the outcome variables: one for the pooled sample, one for male and one for female. Following the regression analyses, we used variance inflation factor (VIF) to ensure that none of the predictor variables were highly associated with each other. Model goodness of fit was assessed with pseudo-*R*^2^ statistics. All statistical tests were two-tailed and a *p* < 0.05 was considered statistically significant for all analyses.

## 3. Results

Sample characteristics were shown in Table 1. The analysis included 2,343 men and women. Female participants comprised more than three-quarter of the sample population (77%) with a great majority of the participants being from rural areas (77%). More than four-fifth reported satisfaction with health (82.4%) and life situation (81.4%) and 77.9% reported being happy. A vast majority expressed improvement in life situation compared with a year ago (81.4%), and nearly all of the participants (95.4%) expect to have better life situation a year later. The percentage of satisfaction about health, life, and happiness was generally higher among those who were 15–19 years, females, currently unmarried, urban residents, located in Demerara-Mahaica region, of East Indian Ethnicity, living in higher wealth quintile households, user of TV/Radio or Internet, and nonsmokers and those who never drank alcohol.

The predictors of reporting satisfaction with health, happiness, and life were presented in Tables 2, 3, and 4, respectively.  Table 2 shows that the odds of satisfaction with health was significantly lower among rural residents [OR=0.600, 95%CI=0.367,0.982] and higher among the mixed race [OR = 0.600, 95%CI = 0.367, 0.982] and higher among the mixed race [OR = 2.294, 95%CI = 1.196, 4.399]. The urban–rural difference was significant only among men [OR = 0.518, 95%CI = 0.297, 0.901], while higher wealth quintiles showed a positive association with health among women only. Smoking or drinking behaviour showed no association with health, but was strongly associated with happiness among women (Table 3). Women living in the rural areas had lower odds of satisfaction with happiness [OR=0.662, 95%CI=0.381,0.845] compared with men. Women having secondary [OR = 0.662, 95%CI = 0.381, 0.845] compared with men. Women having secondary [OR = 2.219, 95%CI = 1.209, 3.720] and higher [OR = 1.600, 95%CI = 1.041, 3.302] education also had higher odds of satisfaction with happiness.  Table 5 indicated that women were significantly less likely [OR = 3.722, 95%CI = 1.502, 9.227] to report life satisfaction compared with men. Rural residency showed an inverse [OR = 0.419, 95%CI = 0.236, 0.873] and abstinence from alcohol [OR = 3.071, 95%CI = 1.659, 6.311] showed a positive association with life satisfaction.

Predictors of life satisfaction in comparison with last year and within next year were presented in Tables 5 and 6, respectively. Similar to life satisfaction at present, women in the rural areas had lower odds of reporting improvement in life situation since last year [OR=0.496, 95%CI=0.155,0.990] and within next year [OR=0.532, 95%CI=0.309,0.989].

TV/radio and Internet users had odds of reporting improvement in life situation within next year.

## 4. Discussion

In the present study we analysed Guyana Multiple Indicator Cluster Survey data to explore the differences in subjective health and well-being related indicators among young adults. As more and more young adults are leaving rural areas attracted by better life and job prospects in urban areas, resource-poor countries like Guyana are facing substantial challenges in providing the same standard of living among both the urban and rural dwellers. As such, rural health is an emerging issue in the areas of development and public health as countries strive to address the growing socioeconomic inequality and improve the quality of life of the underserved people living in the remote areas. The findings of the present study support the concern that the health status of young populations in the rural areas is significantly lower compared with their urban counterparts. However, it is worth noting that the difference was pronounced among women only. This finding is interesting especially because of the fact that women had a significantly higher percentage of reporting satisfaction (or being positive) with all the outcome variables. For instance, more than four-fifth of the women reported being satisfied with health and life, and about three-quarter expect a better life by next year. These findings indicate that the urban-rural difference in subjective health and well-being may not be the same among men and women.

Current evidence on the sex difference regarding health and well-being is generally highly inconsistent and vary widely across countries. However, given the widespread focus in reducing the gender gaps in health well-being, the volume of research on this topic has been increasing both in developing and developed settings. Majority of the surveys on subjective well-being report a marginal difference in health and quality of life between men and women, with women reporting slightly higher subjective well-being than men in some countries [33]. A cross-cultural study using data from World Values Survey (WVS) on 80 countries reported that women are generally happier than men in most African and many developing countries, while men are happier in around 15 European and other industrialized countries [33]. However, a recent meta-analytical study found no significant gender differences in subjective well-being and that greater national gender inequality significantly predicts greater gender differences in job satisfaction, but not life satisfaction [34]. Findings of the present study enrich the prevailing concept that area of residence may well be an important predictor of the sex difference. In brief, our findings suggest that living in rural areas is inversely associated with health among men and with happiness and life satisfaction among women. Exploring the underlying mechanisms behind these nuanced differences goes beyond the scope of the present study as we had data that were cross-sectional. More qualitative and longitudinal studies are required to investigate the pathways through which urbanicity influences health and well-being among the adult population.

It is also worthy of mentioning that women were also significantly less likely to report satisfaction in comparison with last year and expect any improvement in the next year. These findings are hard to interpret in light of the present analysis and warrant for further in-depth studies to delve into the psychosocial and broader determinants of health that may explain the gender differentials in subjective quality of life. The possible mechanism might be the lower socioeconomic status among women, an important roadblock to empowerment and access to resources necessary for ensuring better living conditions. We found that higher households wealth status was significantly associated with higher odds of reporting satisfaction with health and higher education with higher odds of reporting life satisfaction among women. These findings indicate a potentially positive role between women's socioeconomic status and health and well-being. Therefore, it is arguable that investing in these indicators may help promote women's health and well-being and minimise the urban-rural gap as well. Quite surprisingly, having access to electronic media and Internet did not show any association between health and life satisfaction. Electronic media plays an important role in health communication which in turn exerts various health-promoting effects. The absence of any association between the two might indicate a potential ineffectiveness of the media channels in communicating health messages among young adults.

As global population becomes increasingly urbanised, ensuring optimal health and quality of life amid the rapid demographic transition has been a matter of concern for the policy makers in all countries including Guyana. Being one of the least developed countries in South America, Guyana has been struggling to tackle with its development challenges especially unemployment, a burgeoning informal sector, intraurban inequality, and retardation in rural areas [35]. Growing inequalities in health and well-being is unquestionably a major challenge for human development goals and requires evidence-based and action-oriented policy approach for developing sustainable solution. In the current literature, no systematic research evidence is available for gaining a better understanding of the urban-rural disparity in health and well-being of the general population. The single-item tools of measuring well-being, especially happiness and life satisfaction, are becoming increasingly popular among researchers in the developed countries and need to be studied in developing countries as well. These indicators are considered of great relevance for measuring health outcomes such as chronic diseases, evaluating the effectiveness of intervention techniques, as well as for comparing overall living conditions and standard among adult population [36, 37]. From this respect, the findings of the present analysis can be an important contribution to the literature and resource for further studies along this line.

To our knowledge, this is the first study to investigate the urban-rural disparities in subjective health, happiness, and life satisfaction among adult population in Guyana. The findings have important implications for future research and for general practitioners in the primary care settings as well. The current medical care is focused mainly on objective measures such as medical tests and pharmacological interventions, with little to no attention paid to the context the patients are living in. It is recommended that healthcare providers take background information, especially sociocultural and environmental scenarios that might be contributing to the health conditions in the first place. The findings are indicative of regional, such as urban-rural, and social inequalities in the distribution of good subjective health and quality of life especially among women. At policy making level, these disparities should therefore be given appropriate policy actions to address the underlying factors contributing to these unequal health and quality of life outcomes. Apart from the contributions to the literature, our study has several important limitations. Firstly, the data were secondary and we had no control over the selection and measurement of the variables. As a result, we were not able to include a number of relevant factors characteristic of the urban-rural difference in health and healthcare such as environmental condition, access to water and sanitation, public transportation facilities and distance to health facility. Secondly, the sample population was restricted to the age range of 15-49 years and therefore cannot represent the entire population. Thirdly, the variables were self-reported and hence are subject to reporting and recall bias. There is also an overrepresentation of the rural population in the sample, which might have affected the prevalence of health and quality of life. However, the analyses were adjusted for urbanicity to minimise this affect. Last but not least, the data are cross-sectional which precludes making any causal inference between the dependent and explanatory variables. Future studies should focus on a broader range of indicators of subjective well-being specific to the sociocultural environment of Guyana.

## 5. Conclusions

The proportion of young adults reporting satisfaction with their health, happiness, and life is generally good with about four-fifth of the sample population expressing satisfaction on these indicators. However, the proportion varies significantly across some sociodemographic factors, especially place of residence. Living in rural areas was inversely associated with health among men and with happiness and life satisfaction among women. The findings of this study call for policy actions to address the urban-rural gap in health and well-being among young adults in Guyana. More studies are required to investigate the pathways through which urbanicity influences health and well-being among adult population.

## Figures and Tables

**Table 1:** Proportion (%) of sample population reporting satisfaction with health, happiness, and life situation (*n* = 2, 434).

	Sample %	SRH		Happiness		Life satisfaction		Life situation a year ago		Life situation a year after	
		Not satisfied	Satisfied	Not happy	Happy	Not satisfied	Satisfied	Not improved	Improved	Not better	Better
		17.6	82.4	22.1	77.9	22.9	77.1	18.6	81.4	4.6	95.4
*Age *											
15-19	51.4	51.6	50.2	51.3	51.6	52.2	48.7	51.0	51.5	50.5	51.4
20-24	48.6	48.4	49.8	48.7	48.4	47.8	51.3	49.0	48.5	49.5	48.6
*p-value*		*.121*		*.137*		*.071*		*.141*		*.147*	
*Sex*											
Male	23.0	23.7	19.4	22.3	25.3	24.9	16.5	22.3	23.1	20.7	23.1
Female	77.0	76.3	80.6	77.7	74.7	75.1	83.5	77.7	76.9	79.3	76.9
*p-value*		<*.001*		<*.001*		<*.001*		*.069*		<*.001*	
*Currently married*											
No	76.8	75.2	84.9	75.5	80.6	75.2	83.5	82.8	75.3	96.4	75.9
Yes	23.2	24.8	15.1	24.5	19.4	24.8	16.5	17.2	24.7	3.6	24.1
*p-value*		<*.001*		<*.001*		<*.001*		<*.001*		<*.001*	
*Residency *											
Urban	22.7	22.8	32.2	21.9	25.7	23.0	31.7	18.5	23.7	12.6	23.2
Rural	77.3	77.2	67.8	78.1	74.3	77.0	68.3	81.5	76.3	87.4	76.8
*p-value*		*.041*		<*.001*		<*.001*		<*.001*		<*.001*	
*Region *											
Barima-Waini	4.9	4.4	7.0	5.1	4.3	4.6	5.9	8.6	4.0	17.1	4.3
Pomeroon-Supenaam	5.6	5.1	7.9	4.4	9.9	4.4	9.7	7.9	5.1	2.7	5.8
Essequibo Islands-West Demerara	13.9	14.3	12.4	14.0	13.6	14.0	13.8	12.6	14.2	11.7	14.0
Demerara-Mahaica	35.2	34.7	37.4	34.6	37.1	34.9	35.9	34.7	35.3	36.0	35.1
Mahaica-Berbice	6.0	6.4	4.4	6.2	5.4	6.6	4.1	2.9	6.8	4.5	6.1
East Berbice-Corentyne	14.4	15.4	9.8	16.3	7.8	16.5	7.4	8.6	15.7	6.3	14.8
Cuyuni-Mazaruni	5.2	4.9	6.5	5.0	5.8	5.6	3.6	7.3	4.7	4.5	5.2
Potaro-Siparuni	2.9	3.1	2.1	2.9	3.0	2.7	3.8	2.6	3.0	4.5	2.8
Upper Takutu-Upper Essequibo	4.7	4.4	6.1	4.1	6.9	3.4	9.2	9.9	3.5	9.9	4.4
Upper Demerara-Berbice	7.1	7.3	6.3	7.4	6.3	7.3	6.6	4.9	7.7	2.7	7.4
*p-value*		<*.001*		<*.001*		<*.001*		<*.001*		<*.001*	
*Education *											
None	6.0	5.9	6.3	6.4	4.7	6.5	4.3	8.4	5.5	12.6	5.7
Secondary	85.3	86.1	81.3	86.0	82.5	85.5	84.4	85.2	85.3	77.5	85.6
Higher	8.8	8.0	12.4	7.6	12.8	8.0	11.3	6.4	9.3	9.9	8.7
*p-value*		*.048*		*.023*		*.021*		*.023*		<*.001*	
*Ethnicity *											
East Indian	35.1	36.3	29.4	36.9	28.7	37.2	27.8	28.3	36.6	28.8	35.4
African	28.6	29.1	26.6	28.0	30.9	28.9	27.6	25.8	29.3	22.5	28.9
Amerindian	15.2	14.9	16.6	14.5	17.3	13.4	21.2	23.8	13.2	31.5	14.4
Mixed Race	21.1	19.8	27.3	20.6	23.1	20.5	23.3	22.1	20.9	17.1	21.3
*p-value*		<*.001*		<*.001*		<*.001*		<*.001*		<*.001*	
*Wealth index *											
Poorest	17.8	17.9	17.5	18.0	17.1	18.3	16.3	13.2	18.9	14.4	18.0
Second	15.4	14.9	18.0	14.5	18.4	15.2	16.0	10.2	16.6	11.7	15.6
Middle	18.7	18.2	20.8	19.0	17.7	18.8	18.5	15.7	19.4	12.6	19.0
Fourth	28.1	28.2	27.6	28.5	26.6	27.1	31.4	41.7	24.9	46.8	27.2
Richest	20.0	20.8	16.1	20.0	20.1	20.7	17.8	19.2	20.2	14.4	20.3
*p-value*		<*.001*		<*.001*		<*.001*		<*.001*		<*.001*	
*User of TV/Radio*											
Yes	92.2	91.9	93.5	91.9	93.1	92.5	91.0	88.5	93.0	82.9	92.6
No	7.8	8.1	6.5	8.1	6.9	7.5	9.0	11.5	7.0	17.1	7.4
*p-value*		<*.001*		<*.001*		<*.001*		<*.001*		<*.001*	
*Uses internet *											
Yes	65.6	69.8	75.9	63.3	73.6	65.0	67.5	50.6	69.0	42.3	66.7
No	34.4	29.3	24.1	36.7	26.4	35.0	32.5	49.4	31.0	57.7	33.3
*p-value*		<*.001*		<*.001*		*.032*		<*.001*		<*.001*	
*Ever smokes *											
Yes	19.1	18.5	21.5	17.9	23.1	18.6	20.5	22.1	18.4	18.0	19.1
No	80.9	81.5	78.5	82.1	76.9	81.4	79.5	77.9	81.6	82.0	80.9
*p-value*		<*.001*		<*.001*		<*.001*		<*.001*		<*.001*	
*Ever drinks alcohol *											
Yes	63.6	62.3	69.6	61.1	72.1	62.5	67.1	64.0	63.5	55.0	64.0
No	36.4	37.7	30.4	38.9	27.9	37.5	32.9	36.0	36.5	45.0	36.0
*p-value*		<*.001*		<*.001*		*.004*		*.810*		<*.001*	

**Table 2:** Predictors of good SRH among young adult population in Guyana, Guyana MICS 2014.

	Pooled	Men	Women
*Sex (Men)*	1		
Women	2.158	NA	NA
	[0.795, 5.859]		
*Currently married/in union (Yes)*	1	1	1
No	1.616	1.995	1.420
	[0.467, 5.590]	[0.539, 7.384]	[0.395, 5.102]
*Residency (Urban)*	1	1	1
Rural	0.600^∗^	0.518^∗^	0.985
	[0.367, 0.982]	[0.297, 0.901]	[0.161, 6.019]
*Region (Barima-Waini*	1	1	1
*Pomeroon-Supenaam)*	3.394	9.776	2.050
	[0.800, 14.41]	[1.000, 95.57]	[0.905, 4.644]
Essequibo Islands-West Demerara	1.420	4.142	1.858
	[0.341, 5.917]	[0.425, 40.34]	[0.905, 3.815]
Demerara-Mahaica	1.394	3.533	1.180
	[0.378, 5.136]	[0.387, 32.24]	[0.335, 2.970]
Mahaica-Berbice	0.807	2.199	0.752
	[0.159, 4.098]	[0.197, 24.55]	[0.188, 3.003]
East Berbice-Corentyne	1.284	3.592	0.857
	[0.304, 5.422]	[0.359, 35.93]	[0.429, 5.698]
Cuyuni-Mazaruni	1.854	9.268	0.886
	[0.447, 7.682]	[0.918, 93.61]	[0.696, 2.175]
Potaro-Siparuni	1.398	0.925	1.091
	[0.241, 8.113]	[0.0481, 17.77]	[0.507, 2.403]
Upper Takutu-Upper Essequibo	1.389	1.641	0.842
	[0.276, 7.006]	[0.128, 20.97]	[0.270, 1.156]
Upper Demerara-Berbice	0.886	2.340	0.925
	[0.186, 4.222]	[0.211, 25.96]	[0.545, 2.065]
*Education (None)*	1	1	1
Secondary	0.628	0.820	0.780
	[0.226, 1.742]	[0.244, 2.759]	[0.439, 1.490]
Higher	0.427	0.831	0.733
	[0.115, 1.589]	[0.183, 3.768]	[0.462, 1.116]
*Ethnicity (East Indian)*	1	1	1
African	1.419	1.197	2.096
	[0.773, 2.607]	[0.612, 2.344]	[0.492, 4.093]
Amerindian	1.449	1.494	0.608
	[0.547, 3.838]	[0.510, 4.373]	[0.364, 1.735]
Mixed Race	2.294^∗^	2.301^∗^	3.150
	[1.196, 4.399]	[1.110, 4.770]	[0.215, 46.27]
*Wealth quintile (Poorest)*	1	1	1
Second	1.153	1.140	0.236
	[0.554, 2.398]	[0.515, 2.523]	[0.00368, 15.19]
Middle	1.314	1.054	1.854^∗^
	[0.607, 2.845]	[0.451, 2.465]	[1.140, 4.292]
Fourth	0.594	0.412	5.059
	[0.246, 1.433]	[0.147, 1.154]	[0.424, 60.33]
Richest	1.128	0.811	3.184^∗^
	[0.496, 2.567]	[0.314, 2.092]	[2.253, 6.023]
*User of TV/Radio (No)*	1	1	1
Yes	1.170	0.846	10.43
	[0.389, 3.518]	[0.241, 2.975]	[0.416, 261.6]
*User of internet (No)*	1	1	1
Yes	0.701	0.677	1.355
	[0.405, 1.215]	[0.365, 1.255]	[0.124, 14.75]
*Ever smokes (No)*	1	1	1
Yes	1.059	1.073	0.385
	[0.502, 2.233]	[0.368, 3.132]	[0.0775, 1.908]
*Ever drinks alcohol (No)*	1	1	1
Yes	0.741	0.910	0.159
	[0.404, 1.359]	[0.444, 1.864]	[0.0219, 1.153]

Pseudo *R*^2^	0.267	0.189	0.209

Numbers represent odds ratios; 95% confidence intervals in brackets, reference category in () brackets.

^∗^ *p* < 0.05, ^∗∗^*p* < 0.01, ^∗∗∗^*p* < 0.001.

**Table 3:** Predictors of positive estimation of happiness among young adult population in Guyana, Guyana MICS 2014.

	Pooled	Men	Women
*Sex (Men)*	1	NA	NA
Women	1.036		
	[0.431, 2.490]		
*Currently married/in union (Yes)*	1	1	1
No	0.872	0.873	1.368
	[0.288, 2.639]	[0.283, 2.690]	[0.453, 4.135]
			
*Residency (Urban)*	1	1	1
Rural	0.971	0.901	0.662^∗^
	[0.631, 1.493]	[0.565, 1.438]	[0.381, 0.845]
*Region (Barima-Waini*	1	1	1
*Pomeroon-Supenaam)*	0.758	1.061	0.141
	[0.203, 2.835]	[0.220, 5.112]	[0.017, 1.136]
Essequibo Islands-West Demerara	0.739	0.960	0.418
	[0.218, 2.504]	[0.215, 4.283]	[0.165, 3.164]
Demerara-Mahaica	0.850	1.081	0.491
	[0.277, 2.608]	[0.262, 4.456]	[0.165, 4.651]
Mahaica-Berbice	1.144	1.292	1.142
	[0.322, 4.068]	[0.277, 6.028]	[0.808, 2.114]
East Berbice-Corentyne	0.349	0.528	0.441
	[0.0973, 1.249]	[0.112, 2.495]	[0.329, 2.895]
Cuyuni-Mazaruni	0.985	0.697	1.219
	[0.284, 3.412]	[0.127, 3.824]	[0.446, 2.127]
Potaro-Siparuni	1.523	1.127	2.741
	[0.359, 6.452]	[0.193, 6.592]	[0.752, 4.088]
Upper Takutu-Upper Essequibo	2.746	2.420	1.275
	[0.743, 10.15]	[0.503, 11.65]	[0.807, 4.674]
Upper Demerara-Berbice	0.331^∗∗∗^	0.313^∗∗∗^	0.720
	[0.198, 0.554]	[0.176, 0.555]	[0.434, 1.916]
*Education (None/primary)*	1	1	1
Secondary	1.102	0.824	2.219^∗^
	[0.394, 3.082]	[0.270, 2.513]	[1.209, 3.720]
Higher	1.936^∗^	1.430	1.600^∗^
	[1.293, 4.315]	[0.395, 5.170]	[1.041, 3.302]
*Ethnicity (East Indian)*	1	1	1
African	1.276	1.377	0.685
	[0.774, 2.102]	[0.800, 2.372]	[0.437, 1.074]
Amerindian	0.965	1.259	0.597
	[0.404, 2.304]	[0.475, 3.338]	[0.193, 1.847]
Mixed Race	1.261	1.228	0.830
	[0.703, 2.260]	[0.638, 2.362]	[0.0815, 8.457]
*Wealth quintile (Poorest)*	1	1	1
Second	1.288	1.314	1.270
	[0.663, 2.505]	[0.621, 2.780]	[0.123, 13.08]
Middle	1.016	1.216	1.852
	[0.505, 2.047]	[0.558, 2.650]	[0.0978, 35.08]
Fourth	0.805	0.929	0.435
	[0.391, 1.658]	[0.410, 2.105]	[0.0354, 5.331]
Richest	2.100^∗^	2.000	4.187^∗∗^
	[1.121, 3.931]	[0.913, 4.384]	[1.924, 8.609]
*User of TV/Radio (No)*	1	1	1
Yes	0.609	0.377^∗^	1.190
	[0.257, 1.443]	[0.143, 0.993]	[0.588, 2.329]
*User of internet (No)*	1	1	1
Yes	0.450	0.889	1.430
	[0.118, 1.724]	[0.183, 4.334]	[0.807, 2.534]
*Ever smokes (No)*	1	1	1
Yes	0.970	1.260	0.875
	[0.469, 2.003]	[0.552, 2.875]	[0.492, 1.967]
*Ever drinks alcohol (No)*	1	1	1
Yes	1.027	0.848	3.408^∗^
	[0.619, 1.703]	[0.461, 1.560]	[1.387, 9.098]

Pseudo *R*^2^	0.378	0.475	0.376

Numbers represent odds ratios; 95% confidence intervals in brackets, reference category in () brackets.

^∗^ *p* < 0.05, ^∗∗^*p* < 0.01, ^∗∗∗^*p* < 0.001.

**Table 4:** Predictors of life satisfaction among young adult population in Guyana, Guyana MICS 2014.

	Pooled	Men	Women
*Sex (Men)*	1	NA	NA
Women	3.722^∗∗^		
	[1.502, 9.227]		
*Currently married/in union (Yes)*	1	1	1
No	1.622	1.320	2.210
	[0.550, 4.778]	[0.411, 4.236]	[0.518, 9.427]
*Residency (Urban)*	1	1	1
Rural	0.663^∗^	2.130	0.419^∗^
	[0.167, 0.972]	[0.182, 4.199]	[0.236, 0.873]
*Region (Barima-Waini*	1	1	1
*Pomeroon-Supenaam)*	1.763	2.880	2.224
	[0.405, 7.668]	[0.290, 28.63]	[0.0723, 68.38]
Essequibo Islands-West Demerara	0.930	2.234	0.0786
	[0.224, 3.863]	[0.233, 21.41]	[0.00210, 2.935]
Demerara-Mahaica	1.251	2.671	0.704
	[0.336, 4.662]	[0.296, 24.11]	[0.0492, 10.08]
Mahaica-Berbice	0.389	0.566	2.718
	[0.0682, 2.214]	[0.0419, 7.642]	[0.0459, 161.0]
East Berbice-Corentyne	0.450	0.952	1.362
	[0.101, 2.000]	[0.0925, 9.803]	[0.744, 2.495]
Cuyuni-Mazaruni	1.382	1.490	1.444
	[0.720, 2.653]	[0.748, 2.970]	[0.424, 4.924]
Potaro-Siparuni	1.958	1.005	29.30
	[0.346, 11.07]	[0.0516, 19.58]	[0.760, 1129.9]
Upper Takutu-Upper Essequibo	9.037^∗∗^	13.37^∗^	4.518^∗^
	[2.017, 40.49]	[1.378, 129.7]	[1.115, 20.66]
Upper Demerara-Berbice	0.696	1.470	0.511
	[0.149, 3.257]	[0.138, 15.61]	[0.0151, 17.23]
*Education (None)*	1	1	1
Secondary	3.105	4.178	1.322
	[0.663, 14.53]	[0.526, 33.18]	[0.0640, 27.29]
Higher	3.402	3.783	3.801
	[0.628, 18.44]	[0.411, 34.83]	[0.117, 123.6]
*Ethnicity (East Indian)*	1	1	1
African	0.892	0.787	1.097
	[0.506, 1.570]	[0.417, 1.486]	[0.182, 6.595]
Amerindian	0.606	0.795	0.0584
	[0.213, 1.722]	[0.246, 2.573]	[0.00243, 1.406]
Mixed Race	1.182	1.039	0.806
	[0.624, 2.236]	[0.502, 2.149]	[0.127, 5.107]
*Wealth quintile (Poorest)*	1	1	1
Second	1.379	1.557	0.279
	[0.644, 2.950]	[0.643, 3.766]	[0.0330, 2.358]
Middle	1.468	1.696	0.194
	[0.665, 3.241]	[0.683, 4.211]	[0.0183, 2.050]
Fourth	0.914	1.036	0.626
	[0.399, 2.095]	[0.390, 2.752]	[0.0885, 4.432]
Richest	0.665	0.572	0.482
	[0.281, 1.577]	[0.198, 1.651]	[0.0718, 3.236]
*User of TV/Radio (No)*	1	1	1
Yes	0.812	0.714	0.948
	[0.293, 2.255]	[0.203, 2.504]	[0.0885, 10.15]
*User of internet (No)*	1	1	1
Yes	0.629	0.648	0.644
	[0.364, 1.086]	[0.348, 1.207]	[0.425, 1.893]
*Ever smokes (No)*	1	1	1
Yes	0.906	0.708	1.037
	[0.458, 1.790]	[0.277, 1.805]	[0.270, 3.988]
*Ever drinks alcohol (No)*	1	1	1
Yes	1.340	1.177	3.071^∗∗^
	[0.821, 2.186]	[0.682, 2.031]	[1.659, 6.311]

Pseudo *R*^2^	0.404	0.487	0.223

Numbers represent odds ratios; 95% confidence intervals in brackets, reference category in () brackets.

^∗^ *p* < 0.05, ^∗∗^*p* < 0.01, ^∗∗∗^*p* < 0.001.

**Table 5:** Predictors of life satisfaction in comparison with last year, Guyana MICS 2014.

	Pooled	Men	Women
*Sex (Men)*	1	1	
Women	0.991	NA	NA
	[0.417, 2.356]		
*Currently married/in union (Yes)*	1	1	
No	0.597	0.542	0.704
	[0.204, 1.748]	[0.181, 1.625]	[0.397, 1.249]
*Residency (Urban)*	1	1	1
Rural	0.554^∗^	0.614	0.496^∗∗^
	[0.357, 0.796]	[0.328, 1.151]	[0.155, 0.990]
*Region (Barima-Waini*	1	1	1
*Pomeroon-Supenaam)*	3.437	3.127	2.176
	[0.856, 13.80]	[0.601, 16.27]	[0.0758, 62.47]
Essequibo Islands-West Demerara	1.617	1.693	0.0808
	[0.494, 5.299]	[0.395, 7.266]	[0.00304, 2.152]
Demerara-Mahaica	1.713	1.448	0.553
	[0.583, 5.027]	[0.376, 5.574]	[0.0416, 7.340]
Mahaica-Berbice	18.17^∗^	13.43^∗^	1.384
	[1.966, 167.9]	[1.255, 143.7]	[0.381, 5.035]
East Berbice-Corentyne	4.095^∗^	3.677	1.348
	[1.160, 14.45]	[0.803, 16.84]	[0.0504, 36.00]
Cuyuni-Mazaruni	1.245	0.604	3.259
	[0.388, 3.993]	[0.136, 2.679]	[0.321, 33.06]
Potaro-Siparuni	1.854	1.823	1.596
	[0.465, 7.390]	[0.347, 9.564]	[0.0736, 34.64]
Upper Takutu-Upper Essequibo	0.995	0.632	3.619
	[0.295, 3.357]	[0.152, 2.622]	[0.149, 88.01]
Upper Demerara-Berbice	1.407	1.008	0.898
	[0.412, 4.810]	[0.231, 4.397]	[0.0335, 24.11]
*Education (None)*	1	1	1
Secondary	0.599	0.563	0.766
	[0.216, 1.661]	[0.174, 1.820]	[0.0595, 9.853]
Higher	0.733	0.996	0.839
	[0.204, 2.638]	[0.215, 4.625]	[0.0385, 18.31]
*Ethnicity (East Indian)*	1	1	1
African	0.893	0.954	0.736
	[0.501, 1.594]	[0.504, 1.803]	[0.115, 4.718]
Amerindian	1.234	1.268	0.296
	[0.479, 3.176]	[0.440, 3.654]	[0.0177, 4.965]
Mixed Race	0.791	1.020	0.162
	[0.417, 1.501]	[0.494, 2.103]	[0.0221, 1.189]
*Wealth quintile (Poorest)*	1	1	1
Second	1.431	1.527	0.803
	[0.727, 2.817]	[0.715, 3.261]	[0.103, 6.277]
Middle	1.592	1.469	3.557
	[0.776, 3.267]	[0.673, 3.207]	[0.337, 37.54]
Fourth	2.758^∗^	2.264	9.960
	[1.228, 6.192]	[0.933, 5.493]	[0.669, 148.2]
Richest	2.146	2.168	1.505
	[0.977, 4.712]	[0.877, 5.361]	[0.213, 10.62]
*User of TV/Radio (No)*	1	1	1
Yes	1.578	1.495	2.277
	[0.660, 3.774]	[0.541, 4.132]	[0.294, 17.63]
*User of internet (No)*	1	1	1
Yes	0.936	0.905	1.304
	[0.568, 1.544]	[0.518, 1.581]	[0.302, 5.634]
*Ever smokes (No)*	1	1	1
Yes	1.090	1.000	0.764
	[0.668, 1.778]	[0.580, 1.723]	[0.140, 4.162]
*Ever drinks alcohol (No)*	1	1	1
Yes	0.562^∗^	0.675	0.321
	[0.339, 0.931]	[0.373, 1.222]	[0.0852, 1.210]

Pseudo *R*^2^	0.483	0.380	0.302

Numbers represent odds ratios; 95% confidence intervals in brackets, reference category in () brackets.

^∗^ *p* < 0.05, ^∗∗^*p* < 0.01, ^∗∗∗^*p* < 0.001.

**Table 6:** Predictors of life satisfaction within one year, Guyana MICS 2014.

	Pooled	Men	Women
*Sex (Men)*	1	1	
Women	0.474	NA	NA
	[0.223, 1.009]		
*Currently married/in union (Yes)*	1	1	
No	0.937	1.306	0.520
	[0.333, 2.635]	[0.427, 3.988]	[0.185, 1.462]
*Residency (Urban)*	1	1	1
Rural	0.862	0.847	0.532^∗^
	[0.524, 1.420]	[0.488, 1.470]	[0.309, 0.989]
*Region (Barima-Waini)*	1	1	1
*(Pomeroon-Supenaam)*	0.830	0.784	0.711
	[0.243, 2.836]	[0.175, 3.524]	[0.0189, 26.73]
Essequibo Islands-West Demerara	1.291	1.324	0.176
	[0.400, 4.168]	[0.302, 5.802]	[0.00727, 4.235]
Demerara-Mahaica	0.885	0.847	0.122
	[0.305, 2.566]	[0.212, 3.382]	[0.00736, 2.007]
Mahaica-Berbice	1.852	2.506	0.0975
	[0.512, 6.702]	[0.503, 12.49]	[0.00301, 3.156]
East Berbice-Corentyne	2.123	2.277	2.090
	[0.629, 7.170]	[0.494, 10.49]	[0.0638, 68.42]
Cuyuni-Mazaruni	2.739	2.179	2.718
	[0.769, 9.758]	[0.374, 12.70]	[0.264, 28.02]
Potaro-Siparuni	0.666	0.414	1.136
	[0.173, 2.560]	[0.0814, 2.107]	[0.0424, 30.44]
Upper Takutu-Upper Essequibo	1.020	1.594	0.0505
	[0.288, 3.606]	[0.312, 8.154]	[0.00180, 1.420]
Upper Demerara-Berbice	0.599	0.474	0.140
	[0.181, 1.979]	[0.107, 2.096]	[0.00565, 3.493]
*Education (None)*	1	1	1
Secondary	0.641	0.447	4.003
	[0.231, 1.781]	[0.122, 1.641]	[0.261, 61.31]
Higher	0.535	0.397	2.727
	[0.163, 1.751]	[0.0912, 1.725]	[0.111, 67.23]
*Ethnicity (East Indian)*	1	1	1
African	0.725	0.808	0.410
	[0.437, 1.202]	[0.464, 1.407]	[0.0662, 2.544]
Amerindian	0.913	1.064	0.467
	[0.390, 2.138]	[0.399, 2.837]	[0.0245, 8.893]
Mixed Race	0.612	0.882	0.496^∗^
	[0.345, 1.086]	[0.460, 1.691]	[0.304, 0.930]
*Wealth quintile (Poorest)*	1	1	1
Second	1.026	0.937	1.833
	[0.553, 1.902]	[0.467, 1.881]	[0.212, 15.84]
Middle	1.771	1.380	5.107^∗∗^
	[0.893, 3.510]	[0.653, 2.915]	[2.377, 17.42]
Fourth	1.959	1.514	5.885
	[0.969, 3.959]	[0.690, 3.324]	[0.884, 39.19]
Richest	1.763	1.913	1.835
	[0.864, 3.600]	[0.828, 4.419]	[0.273, 12.32]
*User of TV/Radio (No)*	1	1	1
Yes	2.604^∗^	3.058^∗^	3.437
	[1.141, 5.942]	[1.198, 7.805]	[0.374, 31.58]
*User of internet (No)*	1	1	1
Yes	1.921^∗∗^	1.822^∗^	3.912
	[1.189, 3.104]	[1.066, 3.112]	[0.848, 18.03]
*Ever smokes (No)*	1	1	1
Yes	1.392	1.521	0.952
	[0.913, 2.121]	[0.957, 2.419]	[0.225, 4.017]
*Ever drinks alcohol (No)*	1	1	1
Yes	1.468	1.811	0.662
	[0.871, 2.475]	[0.945, 3.468]	[0.172, 2.547]

Pseudo *R*^2^	0.495	0.490	0.366

Numbers represent odds ratios; 95% confidence intervals in brackets, reference category in () brackets.

^∗^ *p* < 0.05, ^∗∗^*p* < 0.01, ^∗∗∗^*p* < 0.001.

## Data Availability

Data used in this study are available through the official website of MICS to registered users.
